# Copper Salts Mediated Morphological Transformation of Cu_2_O from Cubes to Hierarchical Flower-like or Microspheres and Their Supercapacitors Performances

**DOI:** 10.1038/srep09672

**Published:** 2015-04-10

**Authors:** Liang Chen, Yu Zhang, Pengli Zhu, Fengrui Zhou, Wenjin Zeng, Daoqiang Daniel Lu, Rong Sun, Chingping Wong

**Affiliations:** 1 Shenzhen Institutes of Advanced Technology, Chinese Academy of Sciences, Shenzhen, China; 2 School of Materials Science and Engineering, Nanjing University of Posts and Telecommunications; 3 School of Materials Science and Engineering, Georgia Institute of Technology, Atlanta, USA; 4 Department of Electronics Engineering, The Chinese University of Hong Kong, Hong Kong, China

## Abstract

Monodisperse Cu_2_O of different microstructures, such as cubes, flower-like, and microspheres, have been extensively synthesized by a simple polyol reduction method using different copper salts, i.e. (Cu(acac)_2_, Cu(OH)_2_, and Cu(Ac)_2_·H_2_O). The effects of copper salts on the morphology of Cu_2_O were investigated in details through various characterization methods, including X-ray diffraction, transmission electron microscopy, scanning electron microscopy and UV-Vis absorption spectra. The effects of morphology on the electrochemical properties were further studied. Among the different structures, Cu_2_O with the microspheric morphology shows the highest specific capacitance and the best cycling stability compared with those of the other two structures, thus bear larger volume charge during the electrochemical reaction due to the microspheres of small nanoparticles.

Supercapacitor is referred as electrochemical capacitors with the capacity of storing electrical energy in the electrolyte/electrode interface. According to their charge storage mechanisms, they could be divided into three categories, double-layer capacitors (EDLCs), pseudocapacitors, and hybrid capacitors^1^,^2^,^3^,^4^,^5^. Among them, EDLCs show adsorption from the interface of an electrolyte/electrode double layer by electrostatic attraction with accumulation of charges, while pseudocapacitors and hybrid exhibit Faradic redox reactions^1^. Materials like activated carbon, graphite, nanotubes (CNTs), etc., exhibit the capacity as EDLC, and metal oxides, conducting polymers exhibit as pseudocapacitance^2^. Because of their high power density, excellent reversibility, and long cycle life, the development of pseudocapacitor materials is extremely useful for the large-scale applications in automotives and portable electronic systems^3^. Until now, various transition metal oxides, such as RuO_2_, Co_3_O_4_, MnO_2_, Fe_2_O_3_, NiO, V_2_O_5_, CuO, In_2_O_3_, NiO and Cu_2_O, have been investigated as electrode candidates for electrochemical pseudocapacitors^6^,^7^,^8^,^9^,^10^,^11^,^12^,^13^,^14^,^15^,^16^. Among them, Cu_2_O, as a typical p-type semiconductor with a direct band gap of 2.2 eV, has many potential applications in solar cells, electrode materials, sensors, and catalysts^17^,^18^,^19^,^20^. Also, due to the variety and stability of its structure, electrodes made of Cu_2_O usually exhibit remarkable electrochemical performance as supercapacitor. Xue *et al.* recently synthesized hollow octahedra and core@shell structure Cu_2_O by a facile room temperature reaction and their capacitances are 58 F g^−1^ and 88 F g^−1^, respectively^21^. Furthermore, for the electrochemical redox reaction, it usually happens at the interfaces between electrode and electrolyte. So, researchers did a lot of work try to reduce the path of electrons and improve the performance of active materials, such as using the nanosized particles, improving the conductive network of the materials using carbonaceous matrix, and build hollow or hierarchical structures which could accommodating large volume changes or the electrolyte could be quickly permeate into the inner of active materials^22^. Therefore, the morphology and size of Cu_2_O might significantly influence their electrochemistry properties. It is also better to known that the formation of single and hierarchic nanostructures has a wide range of applications, not limited to capacitor performance^22^,^23^,^24^.

Recently, Cu_2_O of cubes, nanowires, solid and hollow spheres, octahedrons, nanoboxes, and multistage shape structures have been prepared through different methods^25^,^26^,^27^,^28^,^29^,^30^,^31^. Among them, the polyol method is most amazing one in which the polyol medium itself acts as both solvent and stabilizer in the process, which can limit the growth of particles and prohibit the agglomeration^32^. Also, in this method, a wide variety of chemical species, including polymers, anions, surfactants, and biomolecules, could be used to control the morphology and size of Cu_2_O, by certain species preferentially adsorbing on the specific crystal surfaces and inhibiting the growth rate^33^,^34^,^35^. For example, Cu_2_O nanospheres with a size of 50–70 nm were synthesized by dissolving Cu(NO_3_)_2_ and PVP in ethylene glycol (EG) with continuous stirring. The main driving force of this reaction is the oxidizing power of NO_3_^-^ and the reducing power of EG^36^. It was also reported that various nanocrystalline Cu_2_O structures were obtained by using organic solution phase method and systematic manipulation of the reaction conditions. In this method, EG was used as the solvent while SDS and Tween 80 were used as surfactants^37^. However, there are fewer reports on using diethylene glycol as both solvent and reducing agent to prepare Cu_2_O particles with various structures.

Herein, monodisperse Cu_2_O with cubic, flower-like and hierarchical microspheric structures were prepared in the modified polyol method using different copper sources, including Cu(acac)_2_, Cu(OH)_2_, and Cu(Ac)_2_·H_2_O. The growth mechanisms of different morphology were discussed in details. Furthermore, the influences of morphology on the electrochemical performances of Cu_2_O supercapacitor anodes were comprehensively studied. Among these three structures, the microspheres show higher specific capacitance and better cycling stability than those of the other two structures.

## Results and Discussion

### Structure and morphological analysis

**Figure 1:**
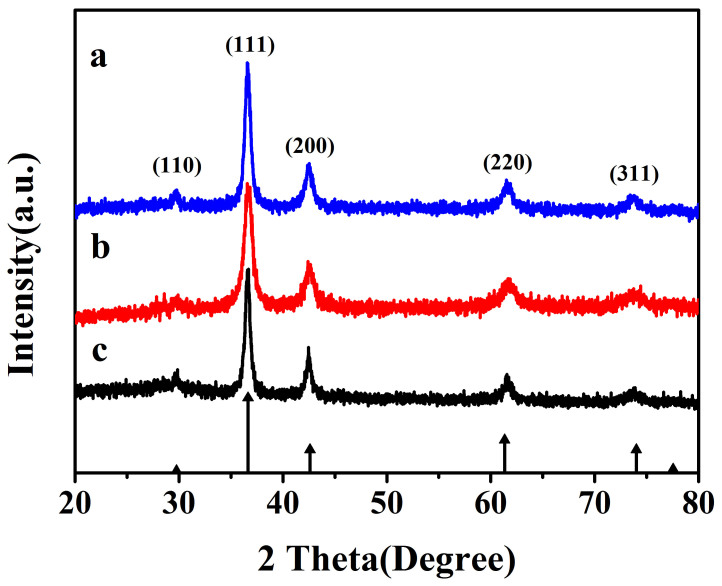
XRD patterns of the Cu_2_O prepared using different copper sources: (a) Cu(acac)_2_; (b) Cu(OH)_2_; (c) Cu(Ac)_2_·H_2_O.

The XRD patterns of the samples synthesized using different copper sources are shown in Figure 1. The XRD data of the three as-synthesized samples are all in good agreement with those of Cu_2_O (JCPDS NO. 65–3288), the six typical peaks located at 29.60°, 36.52°, 42.44°, 61.53°, 73.68° and 77.74°, which were attributed to the (110), (111), (200), (220), (311) and (222) planes of cuprous oxide, respectively. No characteristic peaks arising from Cu or CuO could be observed in the XRD patterns, indicating that the products obtained via our synthetic routes consist of only Cu_2_O phase. From the XRD results, it reveals that pure Cu_2_O could be successfully obtained using these three kinds of copper sources.

**Figure 2:**
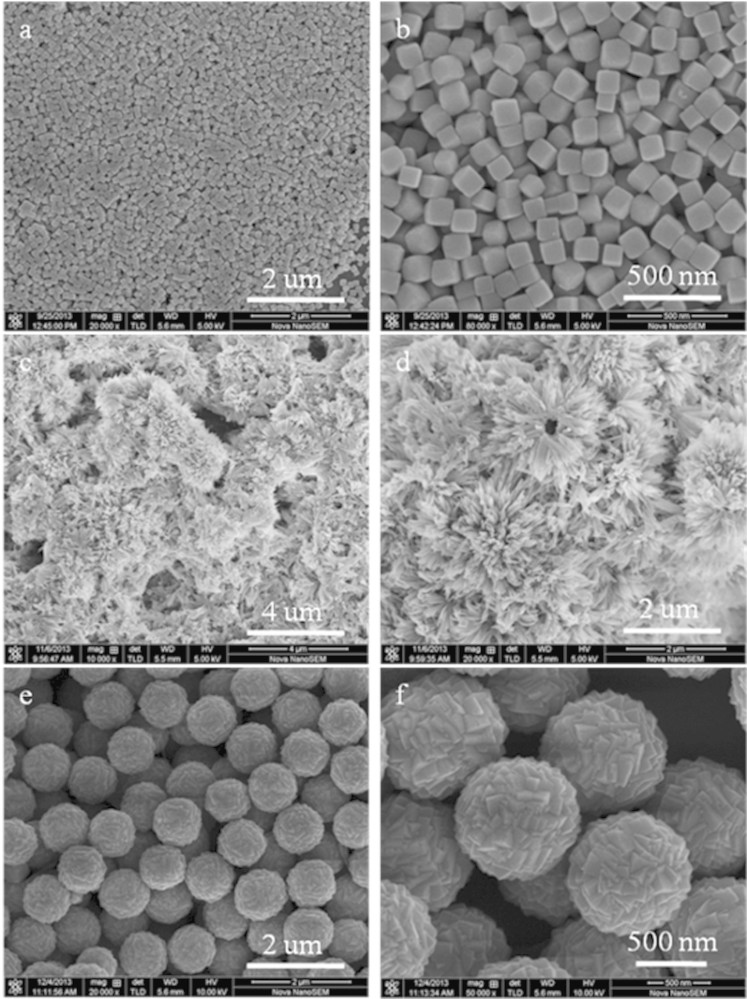
SEM images of the Cu_2_O prepared using different copper sources: (a and b) Cu(acac)_2_; (c and d) Cu(OH)_2_; (e and f) Cu(Ac)_2_·H_2_O.

**Figure 3:**
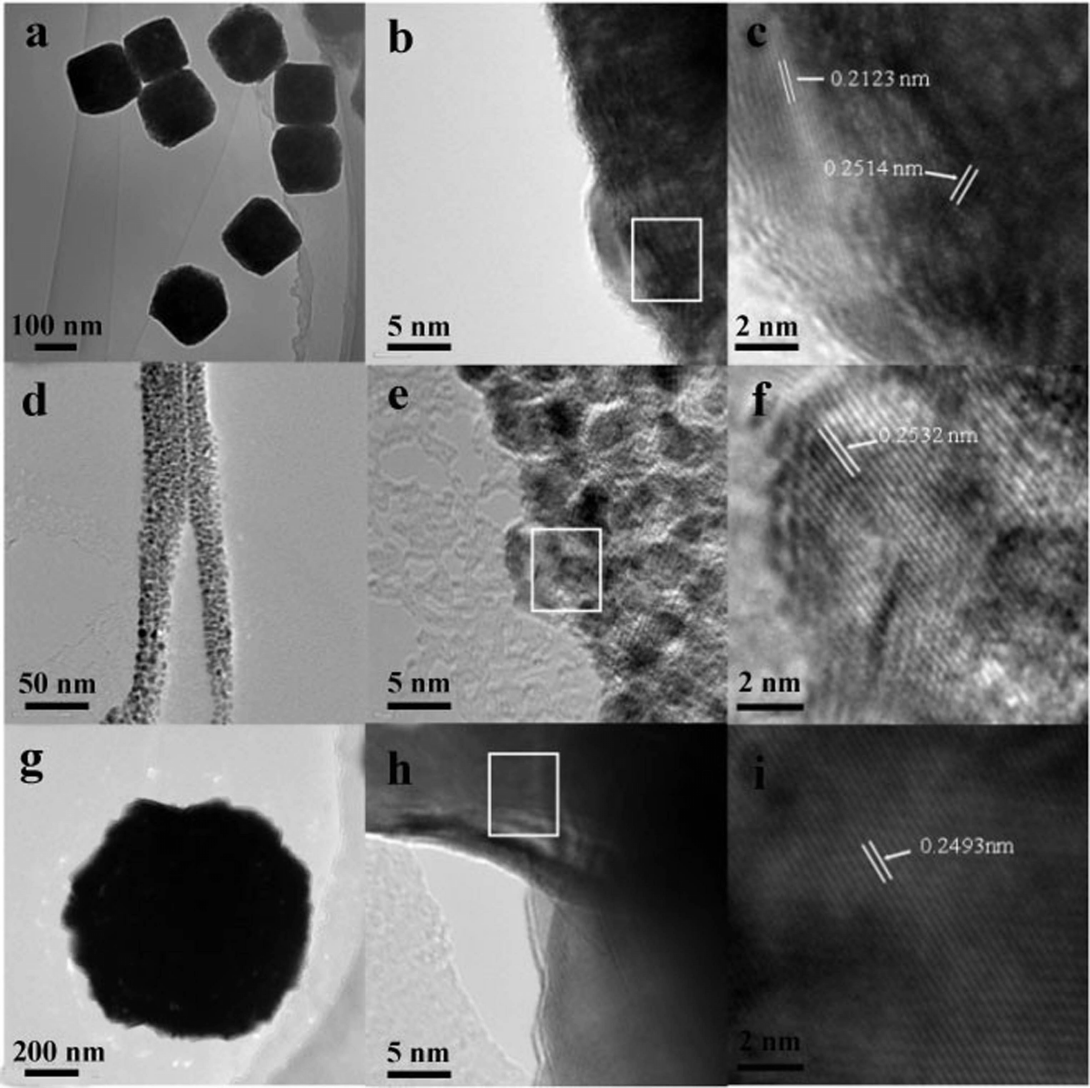
TEM images of Cu_2_O prepared using different copper sources: (a and b) Cu(acac)_2_; (d and e) Cu(OH)_2_; (g and h) Cu(Ac)_2_·H_2_O; (c, f and i are the corresponding HRTEM image of Cu_2_O circled with a square in the TEM images).

SEM was employed to investigate the size and morphology of Cu_2_O synthesized using different copper salts and the results are shown in Figure 2. As shown in Figure 2a, the Cu_2_O synthesized using Cu(acac)_2_ has a cubic shape with a narrow size distribution, the average width of the cubes is about 120 nm. From the magnified SEM image (Figure 2b), it can be clearly observed that the surfaces of these Cu_2_O cubes are very smooth with clear distinction of the edges and corners. When using Cu(OH)_2_ as the precursor, it is clear that the sample presents hierarchical flower-like structure which was composed of many nanowires with a diameter of ~25 nm and a length of ~800 nm (Figure 2c and 2d). Figure 2e is the panorama image of the Cu_2_O prepared by choosing Cu(Ac)_2_·H_2_O as the copper source. It shows that it comprises nearly monodisperse particles with a spherical morphology, averaging 1 μm in diameter. From the high-resolution image of Figure 2f, it can be seen that the microspheres are made up of closely packed prismatic bulge-like structures. The above results indicated that the morphology of the final Cu_2_O samples could be regulated by simply changing the copper sources while keeping the other experiment parameters same. Figure 3 shows the TEM and HRTEM images of the as-synthesized Cu_2_O with cubic, flower-like and hierarchical microspheric structures. Figure 3a and 3b show clearly that the surface of cubic-like Cu_2_O particles are rather smooth which is consistent with the SEM results. The interplane distances of 0.2123 nm and 0.2514 nm calculated from HRTEM of the nanocubes (Figure 3c) are well consistent with the interplane distance values of (200) and (111) faces in the cubic Cu_2_O^20^. As for Cu_2_O made from Cu(OH)_2_, the whole flower-like structure is too big, here only presents the TEM images of its secondary structure-nanowires. It is interesting to note that the sub-nanowire composed of small nanoparticles in the range of 5–10 nm are in intensive arrangement (Figure 3d and 3e). Fringes with spacing of *ca.* 0.2532 nm taken from the small nanoparticles are corresponding to the (111) plane of Cu_2_O (Figure 3f). Figure 3g and 3h display the TEM images of an individual Cu_2_O microsphere which has rather rough surface corresponding to the bulges shown in the SEM images. Also, the pure black microsphere indicates the particle is solid and not the inner hollow or core-shell structure. The observed width, 0.2493 nm, of the adjacent lattice fringes corresponds to the (111) plane of Cu_2_O. The surface area of the Cu_2_O with different morphology were characterized by the nitrogen adsorption-desorption isotherm measurements, and their corresponding BET surface area is 4.1 m^2^ g^−1^, 27.5 m^2^ g^−1^ and 4.3 m^2^ g^−1^ for the cubes, flower-like and microspheres, respectively.

### Formation mechanism

As discussed above, Cu_2_O with cubic, flower-like and hierarchical microspheric structures were obtained only by simply changing the copper salts in the normal polyol method. And it is well-known that the copper salts could be reduced by diethylene glycol (DEG) to form Cu_2_O, which are widely used in the synthesis of Cu_2_O with different morphologies^17^,^18^,^27^. The chemical reactions are as follows:
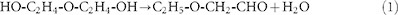

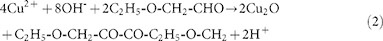


**Figure 4:**
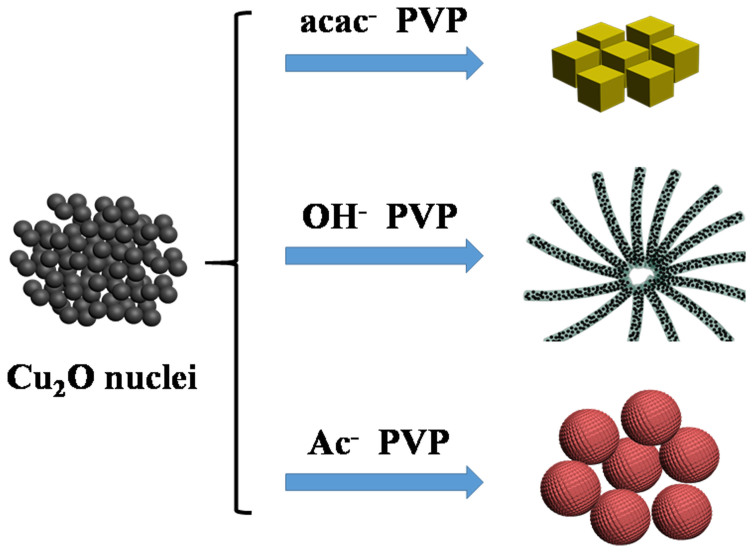
Schematic illustration of the Cu_2_O with disparate morphologies resulting from different copper sources.

At the beginning of the reaction, Cu^2+^ is reduced by the decomposition product of DEG at high temperature (Equation (1) and (2)) and instantly formed the spherical Cu_2_O nanocrystalline. The surface of the new generated Cu_2_O nanocrystalline contains high index crystallography planes, through which the particles tend to aggregate with each other to decrease the surface energy of the planes^1^. As the reaction proceeding, the polyhedral particles would grow along different directions with different growth rates due to their different surface energies. According to the previous reports^38^, the growth rate ratio *R* of the {100} and {111} directions usually determines the geometry of the Cu_2_O crystals. That is, when *R* = 0.58, cubic like Cu_2_O will be prepared. Therefore, the formation of our cubic Cu_2_O might be caused by the different adsorption of acac^-^ and PVP on the surface of (111) and (100), which leading to the different growth rates of these two crystal faces. In the present system, we got the Cu_2_O hyper polyhedral microspheres composed of prismatic structures through changing the copper source to Cu(Ac)_2_·H_2_O. Thus, we inferred that the adding of Ac^-^ can lead to stronger interaction with the (111) facet than acac^-^. This may result in the increasing of *R*, the value of which can be speculated higher than 1.73 due to the hyper polyhedron structure of our product^1^. While flower-like Cu_2_O composed of nanowires were obtained by changing copper source to Cu(OH)_2_. It is known that Cu(OH)_2_ could be thermal decomposed into tiny CuO particles at high temperature (~80°C). Therefore the formation of nanowires might be interpreted that the CuO particles once generated is rapidly reduced to Cu_2_O nanoparticles, at the same time, organics, such as excessive polyols and PVP, coated on the Cu_2_O nanoparticles to form the nanowires. Namely, the synergistic effect between the pyrolysis of Cu(OH)_2_, the instant reduction of CuO and the coating of organics, together create the flower-like Cu_2_O assembling by sub-nanowires. In conclusion, the diverse copper sources possessing different anions used in this reaction process play a critical role on the morphology of the resulting Cu_2_O. Also, the effect of DEG acted as both solvent and reducing agent is indispensable. These factors synergistically lead to the formation of Cu_2_O with disparate morphologies. The simple sketch of these three reaction processes are depicted in Figure 4.

**Figure 5:**
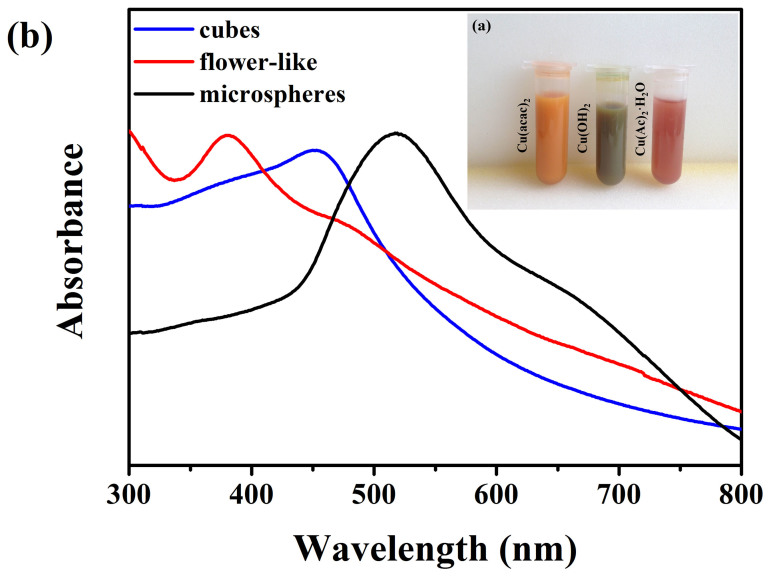
(a) The photos of the final mixtures containing Cu_2_O particles synthesized using different copper sources; (b) UV-Vis spectra of different morphology of Cu_2_O dispersed in ethanol.

A series of color variations have been observed during the reactions which could be used to monitor the reaction process. In addition, as shown in Figure 5a, the colors of the mixture containing the final Cu_2_O particles are entirely distinct. They present orange, green and red brown in turn when using Cu(acac)_2_, Cu(OH)_2_, and Cu(Ac)_2_·H_2_O as the copper source respectively. The colors of the final reaction mixtures might have relationship with the size and morphology of the Cu_2_O. To confirm this, the UV-Vis absorption spectra were taken with the Cu_2_O particles dispersed in ethanol (Figure 5b). In general, the optical absorption peak of bulk Cu_2_O is at 570 nm (band gap ~2.17 eV)^39^,^40^. The Cu_2_O cubes exhibit absorption peak located at 470 nm, the blue shift compared with the bulk Cu_2_O might be due to the size effect. For flower-like Cu_2_O synthesized using Cu(OH)_2_, the broad absorption peak is from 380 nm to 500 nm, resulting from the inhomogeneous size of wires and the flower-like structure. As Luo has reported^41^, Cu_2_O with the similar flower-like structure has a centered peak at 370 nm, giving absorption edge energies corresponding to *E_g_* = 2.23 eV and the increase in the band gap of the Cu_2_O nanoflowers possibly be the resulting of the quantum confinement effects arising from the tiny petals and secondary small nanoparticles. Then, the plasma absorption peak of Cu_2_O with the hierarchical microspheric structure is shown at 518 nm probably resulting from the larger size and rough surface.

### Electrochemical performance

**Figure 6:**
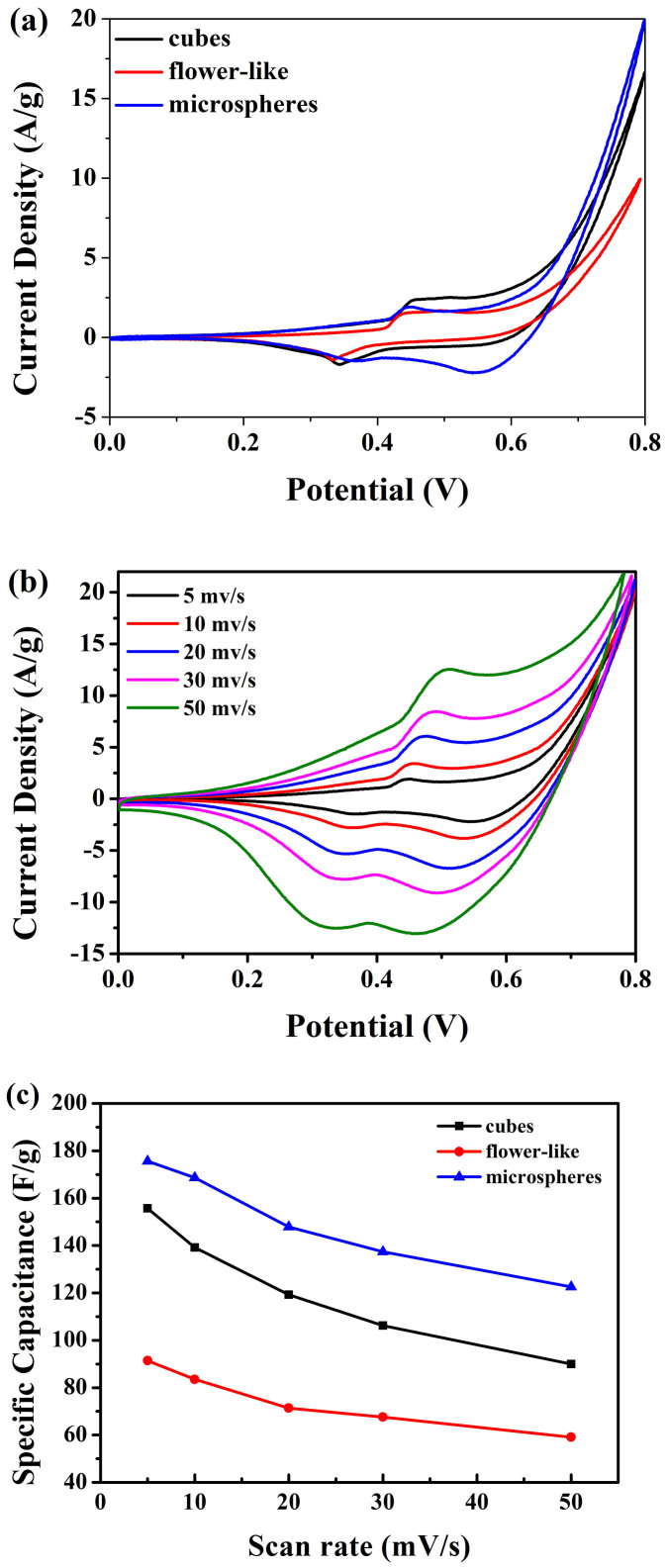
(a) CV curves of cubes, flower-like and microsphere structures in 2 M KOH aqueous solution at scan rates of 5 mV s^−1^. (b) CV curves of microspheres at different scan rates of 5, 10, 20, 30 and 50 mV s^−1^ in 2 M KOH solution. (c) Specific capacitance of the three structures recorded at various potential scan rates.

The electrochemical measurements of the three Cu_2_O particles with different morphology were performed with a three-electrode system in 2 M KOH solution. Figure 6a displays the CV curves of the as-synthesized cubic, flower-like, and microspheric morphology in the potential range of 0 to 0.8 V (*vs*. Hg/HgO) at a potential scan rate of 5 mV s^−1^. For each curve, a typical pair of anodic and cathodic signals and a broad redox background is clearly visible, indicating that the electrochemical mechanism is governed by pseudocapacitive behavior. This behavior differs remarkably from the electric double-layer capacitance, which would produce a CV curve of nearly ideal rectangular shape. The pseudeocapacitance of the Cu oxide electrode is the result of transitions between oxidation states Cu(I) oxide-Cu(II) oxide and vice versa. The redox reaction for the Cu oxide electrode is presented as follows:^42^
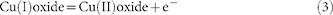


It is very clear that the areas surrounded by the CV curves of the microsphere electrode are larger than those of the cubes and flower-like electrodes at the same scan rate, indicating a higher specific capacitance of the microsphere electrode. The reasons why Cu_2_O microspheres has the best specific capacitance were analysed. For the electrochemical peaks are consistent with the discharge-charge plateaus in Figure 6a, in the cycle of microsphere structure, the two cathodic peaks are observed at 0.36 and 0.54 V, corresponding to the multistep electrochemical Cu^+^ reaction process or additional sites for Cu^+^ intercalation. Meanwhile, in the cycle of cubic and flower-like structures, the decrease of the individual peak intensity and integral area resulting in reversible losses, is observed with shifts to 0.33 and 0.58 V of the peak potentials in the cathodic direction. So it indicates that the rough surface of microspheres could endure large volume charge during the electrochemical reactions and show the highest specific capacitance.

The specific capacitance calculated from the CV curves can be derived from the Equation (4):
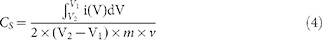


Where *C_s_* is the specific capacitance from evaluated samples; *V_1_* and *V_2_* are the starting and ending points of potential window, respectively; *i(V)* is the instantaneous current as the function of potential; *m* is the mass of two symmetric devices; and *v* is the scan rate in mV s^−1^. The calculated values of specific capacitance at 5 mV s^−1^ scan rate are 157.5 F g^−1^, 92.3 F g^−1^ and 173.2 F g^−1^ for cubes, flower-like and microsphere of Cu_2_O electrode, respectively. It is seen that, microspheres exhibit the maximum specific capacitance as stated above.

Figure 6b shows the CV curves of the microsphere structures recorded at various potential scan rates. Pairs of well-defined cathodic and anodic signals are clearly observed over the entire range of scan rates from 5 to 50 mV s^−1^. The enhancement in current with the scan rate suggests the effective utilization of electrode material by the electrolyte, owing to better ionic diffusion, mostly as a result of well-spaced nanostructured, indicating an ideally capacitive behaviour^43^,^44^. The shape of current responses is essentially the same over the entire range of scan rates, indicting rapid faradaic reactions at large scan rates. As shown in Figure 6c, the optimum specific capacitance of the microsphere evaluated at 50 mV s^−1^ is 122.6 F g^−1^; as much as 70% of the capacitance can be maintained relative to that measured at 5 mV s^−1^ (173.2 F g^−1^). In contrast, the cubic structures retained only 57% of the initial capacitance when the potential scan rate was increased by the same amount. This rapid charge-discharge is an attractive feature for high-power supercapacitor applications, which are essential for rapid applications of energy such as in automotives.

**Figure 7:**
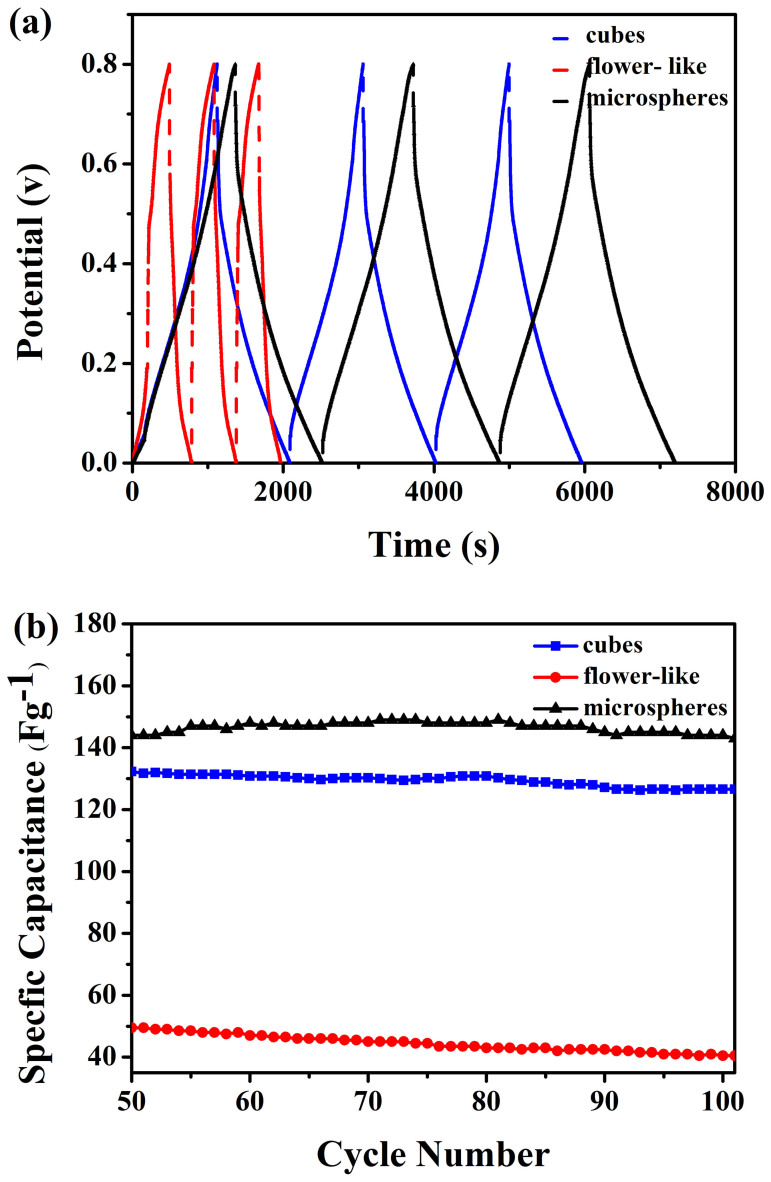
(a) Galvanostatic charge/discharge curves of the last 3 cycles out of 100 cycles at a current density of 0.1 Ag^−1^ of flower-like, cubes and microsphere structures. (b) Cycling performance of the Cu_2_O with different morphology at a current density of 0.1 A g^−1^.

The galvanostatic charge/discharge curves of the cubes, flower-like, and microsphere structures at current density of 0.1 A g^−1^ are depicted in Figure 7a, and their cycle stabilities at the same current density are presented in Figure 7b, respectively. As we can see from Figure 7a, the charge/discharge curves are nearly symmetric with slight curvature. These charge/discharge curves can be employed to estimate the average specific capacitances (*C_s_*) of the as-obtained cubic, flower-like, and microspheric morphology from the following equation (5):
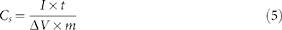
Where *I* is the constant discharge current, *t* is the discharge time, *m* is the mass of the active material on the electrode, and *ΔV* is the potential window. The specific capacitance value of the microsphere Cu_2_O is calculated to be about 144 F g^−1^ at discharge current density of 0.1 A g^−1^, and the cubic Cu_2_O has the nearly specific capacitance value of about 132 F g^−1^ at the same discharge current density. However, the specific capacitance value of Cu_2_O flower-like structure at the discharge current density of 0.1 A g^−1^ catches the lowest value of about 45 F g^−1^. As shown in Figure 7b, the columbic efficiency of these three structures show the similar efficiency of about 99% in the 100 cycles and the specific capacitances of flower-like structure, cube and microsphere decrease from 45 F g^−1^ to 30 F g^−1^, 132 F g^−1^ to 126 F g^−1^ and 144 F g^−1^ to 143 F g^−1^ at the discharge current density of 0.1 A g^−1^ after 100 cycles, respectively. The results further prove that the Cu_2_O microspheres have much predominance as electrochemical energy storage materials and better cycling stability than that of Cu_2_O cubes and flower-like structures. The reason for the highest capacitance and cycling performance of microsphere morphology might be caused by the uniform structure with rough surface which could endure large volume charge during electrochemical reactions. What's more, the microspheres composed of many small particles increase the electrical properties. On the contrary, small crystallites embedded in an amorphous matrix form nanowires, which self-assembled to flower-like structure seriously hinder the interaction between the Cu_2_O particles gain the lowest specific capacitance value of about 45 F g^−1^. The smooth surface of uniform cubic Cu_2_O obtains the specific capacitance just under 12 F g^−1^ of microspheric Cu_2_O.

## Conclusions

In summary, we successfully synthesized series of monodisperse Cu_2_O with various structures through different copper sources by a facile polyol method. The obtained Cu_2_O with different morphology is resulting from the disparate copper salts possessing different anions, acac^-^, OH^-^, and Ac^-^, which could selectively adsorbed on the various crystal face of Cu_2_O and leading to the diverse growth rates of each crystal direction. Furthermore, the effects of shape and size on the electrochemical properties of Cu_2_O were investigated. It shows that the Cu_2_O with hierarchical microspheres has much predominance as supercapacitor materials than that of Cu_2_O with cubic and flower-like structures. The specific capacitance value of the Cu_2_O microspheres is calculated to be about 144 F g^−1^ at discharge current density of 0.1 A g^−1^. The microspheres also present higher specific capacitance and better cycling stability than that of the other structures, because that the rough surface of uniform microspheres composed of small particles can endure large volume charge during electrochemical reactions. It also provides a simple method to prepare Cu_2_O with cubic or flower-like nanostructures, which might find applications in other fields, such as gas sensors, CO oxidation catalysts, and various heterogeneous catalysts.

## Experimental Section

### Material synthesis

Copper hydroxide (Cu(OH)_2_) and copper acetylacetonate (Cu(acac)_2_) were purchased from Aladdin Reagent Co. Ltd. Diethylene glycol (DEG), polyvinyl pyrrolidone (PVP, K-30) and copper acetate monohydrate (Cu(Ac)_2_·H_2_O) were purchased from Sinopharm Chemical Co. Ltd.

In a typical synthesis, 0.01 mol of Cu(acac)_2_ and 2 g PVP were mixed into 100 mL DEG inside a round-bottom flask and keep vigorously stirring from room temperature (RT) to 170°C, then the mixture was stirred at 170°C for 30 min till the formation of Cu_2_O we needed. The resulting dark yellow precipitate was collected by centrifugation, washed with pure ethanol, and finally dried under vacuum at RT for 12 h. For comparison, Cu_2_O with other morphology were prepared under the same experiment procedure except changing the copper source from Cu(acac)_2_ to Cu(OH)_2_ or Cu(Ac)_2_·H_2_O, only shorten the reaction time to 20 min as using Cu(OH)_2_ or prolong the reaction time to 1 h when choosing Cu(Ac)_2_·H_2_O.

### Characterization

X-ray diffraction (XRD) patterns of the samples were recorded on an X-ray diffractometer (Rigaku D/Max 2500, Japan) using the K*α* radiation of Cu (*λ* = 1.54187 Å) from 20° to 80° at a scanning rate of 8°·min^−1^. The nanoscopic feature of the samples was observed by field-emission scanning electron microscopic (FE-SEM, FEI Nova Nano SEM 450) and transmission electron microscopy (TEM, FEI Tecnai G2F20S-TWIN). The UV-Vis absorption spectra of the samples were recorded on an UV-*Vis*-NIR spectrometer (Shimadzu UV-3600, Japan) with a wavelength range of 300–800 nm. Nitrogen adsorption–desorption isotherms for surface area were measured using a Micromeritics ASAP 2020 BET apparatus.

Nitrogen adsorption–desorption isotherms for surface area were measured using a Micromeritics ASAP 2020 BET apparatus with liquid nitrogen at 77 K.

### Electrochemical measurements

The electrochemical measurements were conducted using a three-electrode mode in a 2 M KOH aqueous solution. The working electrode was prepared by mixing Cu_2_O, acetylene black and polytetrafluoroethylene (PTFE) in a weight ratio of 70:20:10. Briefly, the resulting paste was pressed on a sheet of nickel foam at 10 MPa and the surface area of the electrode equal to the area of nickel foam, which is 1.766 cm^2^. The amount of active materials was totally about 10.00 mg, by the same coating method. The mercuric oxide electrode was used as the reference electrode, and the Pt wire as a counter electrode. The cyclic voltammetry (CV) was carried out on a Zahner Zennium electrochemical workstation. Charge-discharge cycling tests were carried out between 0 and 0.8 V on a Land CT2001 battery test system at room temperature.

## Author Contributions

L.C. performed the experiment, Y.Z. and P.Z. assisted the experiments. F.Z. did the TEM and SEM, P.Z. designed the research. W.Z. modifed the english and also help do the BET test. D.L., R.S. and C.W. conceived the study. L.C. wrote the manuscript. All authors discussed the results on the manuscript and reviewed the manuscript.
